# Supplementary material for: Evaluating the Removal of the Antibiotic Cephalexin from Aqueous Solutions Using an Adsorbent Obtained from Palm Oil Fiber
Source: Molecules. 2021 Jun 2;26(11):3340. doi: 10.3390/molecules26113340 (PMC8199501; doi:10.3390/molecules26113340)
Supplement: Supplementary file 1 [file molecules-26-03340-s001.zip › molecules-1179655-supplementary.pdf]

Supporting information

Evaluating the removal of the antibiotic cephalexin from aqueous solutions using an adsorbent obtained from palm oil fiber

Nancy Acelas[a]*, Sandra M. Lopera[b], Jazmín Porras[c] and Ricardo A. Torres-Palma[b]

[a]Grupo de Materiales con Impacto, Mat&mpac. Facultad de Ciencias Básicas, Universidad de Medellín, Medellín, Colombia.

[b]Grupo de Investigación en Remediación Ambiental y Biocatálisis (GIRAB), Instituto de Química, Facultad de Ciencias Exactas y Naturales, Universidad de Antioquia UdeA, Calle 70 No. 52-21, Medellín, Colombia.

[c]Grupo de Investigaciones Biomédicas Uniremington, Facultad de Ciencias de la Salud, Corporación Universitaria Remington (Uniremington), Calle 51 No. 51-27, Medellín, Colombia.

*Corresponding authors

E-mail address: nyacelas@udem.edu.co (Nancy Acelas)

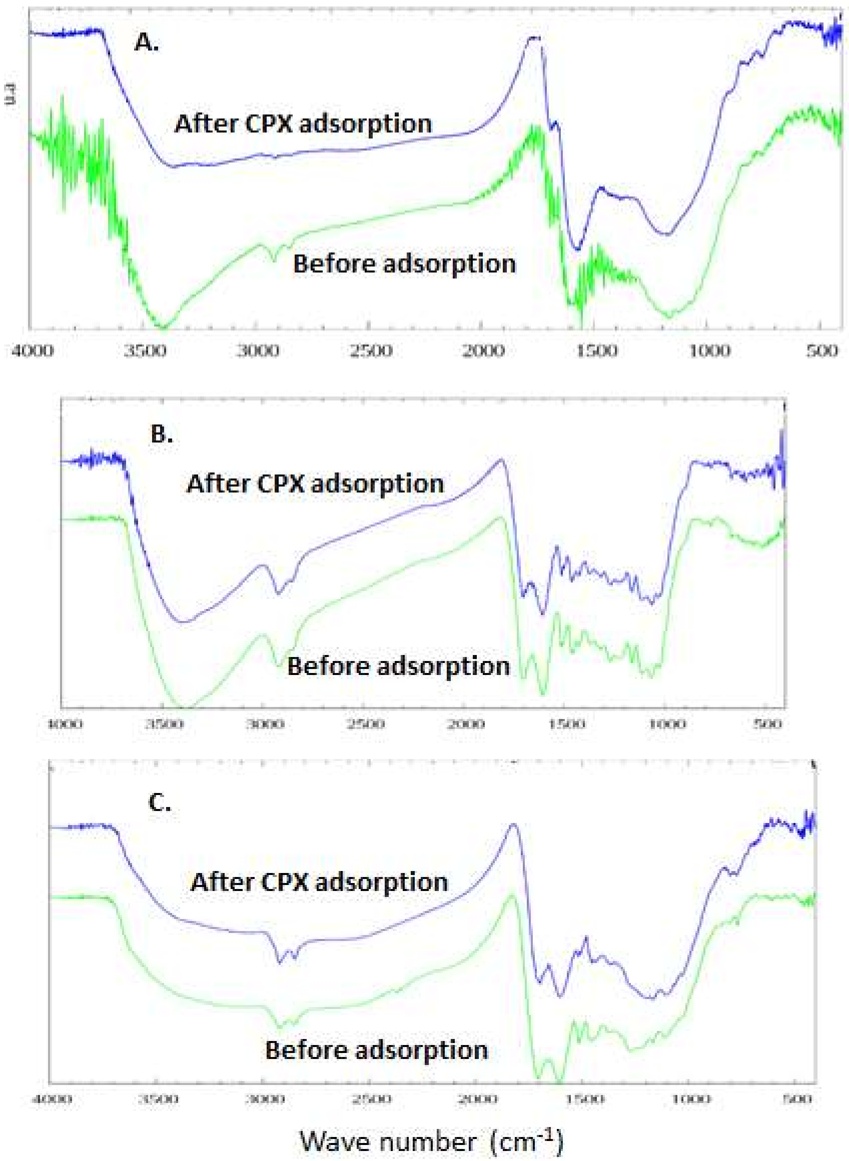

Figure S1. FTIR spectrum for adsorbent materials before and after CPX removal A. F_{Zn}; B. F_{HA}; C. S_{Zn}

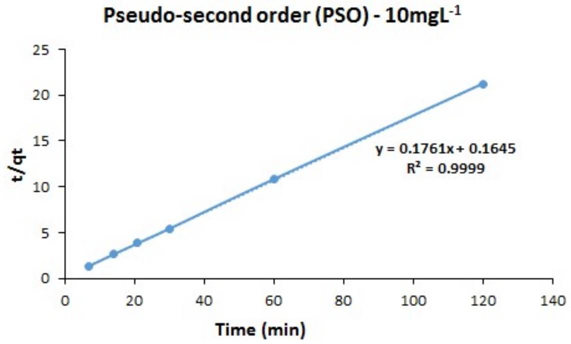
Pseudo-second order (PSO) - 10mgL-1
t/qt
Time (min)
y = 0.1761x + 0.1645
R² = 0.9999

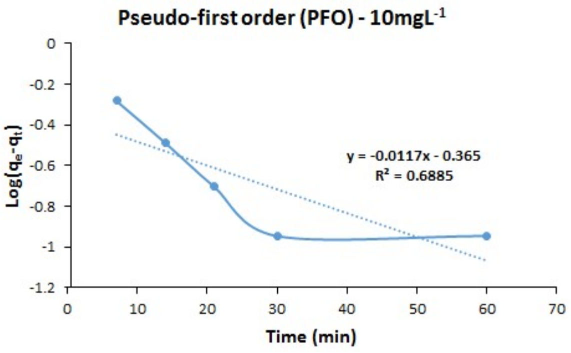
Pseudo-first order (PFO) - 10mgL-1
Log(qe-qt)
Time (min)
y = -0.0117x - 0.365
R² = 0.6885

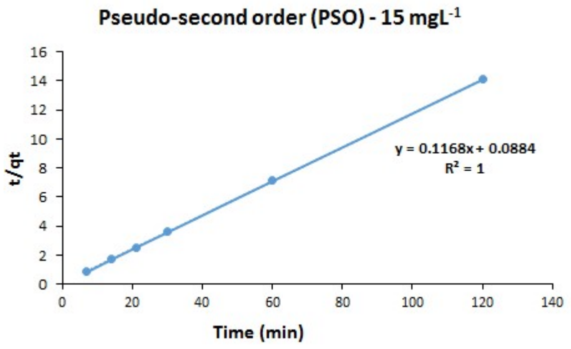
Pseudo-second order (PSO) - 15 mgL-1
t/qt
Time (min)
y = 0.1168x + 0.0884
R² = 1

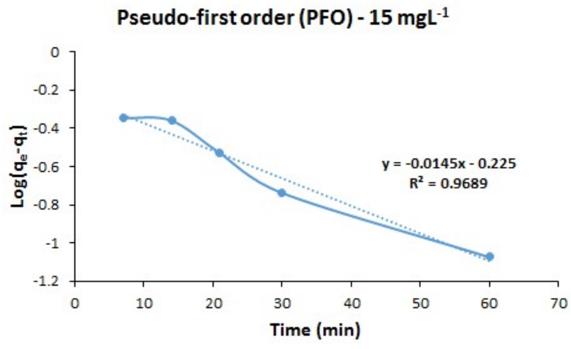
Pseudo-first order (PFO) - 15 mgL-1
Log(qe-qt)
Time (min)
y = -0.0145x - 0.225
R² = 0.9689

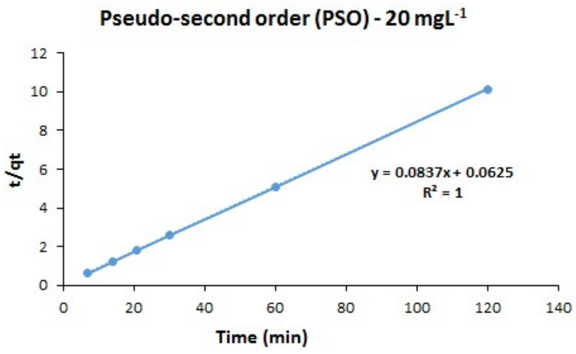
Pseudo-second order (PSO) - 20 mgL-1
t/qt
Time (min)
y = 0.0837x + 0.0625
R² = 1

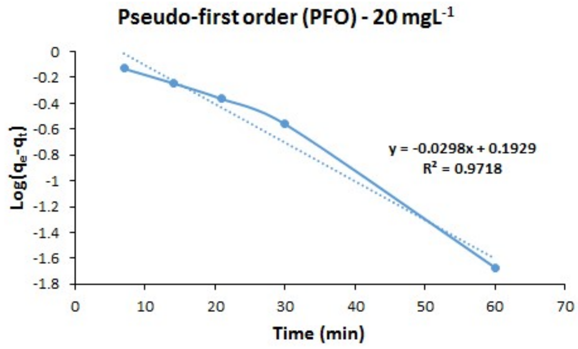
Pseudo-first order (PFO) - 20 mgL-1
Log(qe-qt)
Time (min)
y = -0.0298x + 0.1929
R² = 0.9718

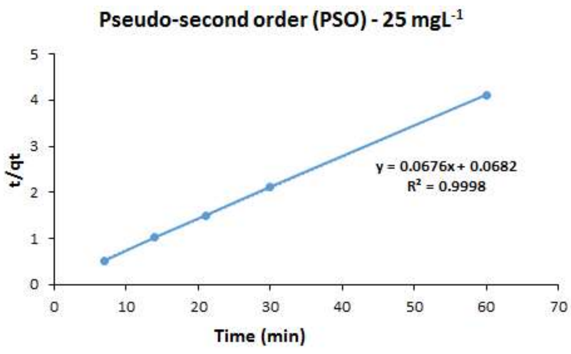
Pseudo-second order (PSO) - 25 mgL-1
t/qt
Time (min)
y = 0.0676x + 0.0682
R² = 0.9998

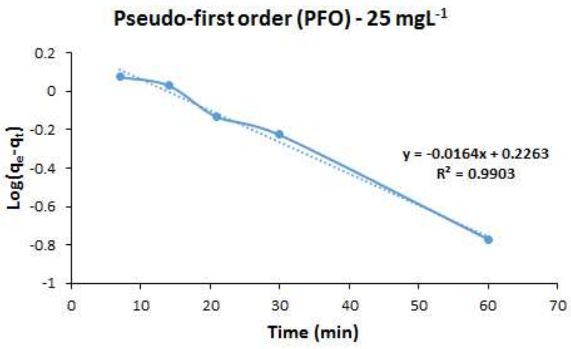
Pseudo-first order (PFO) - 25 mgL-1
Log(qe-qt)
Time (min)
y = -0.0164x + 0.2263
R² = 0.9903

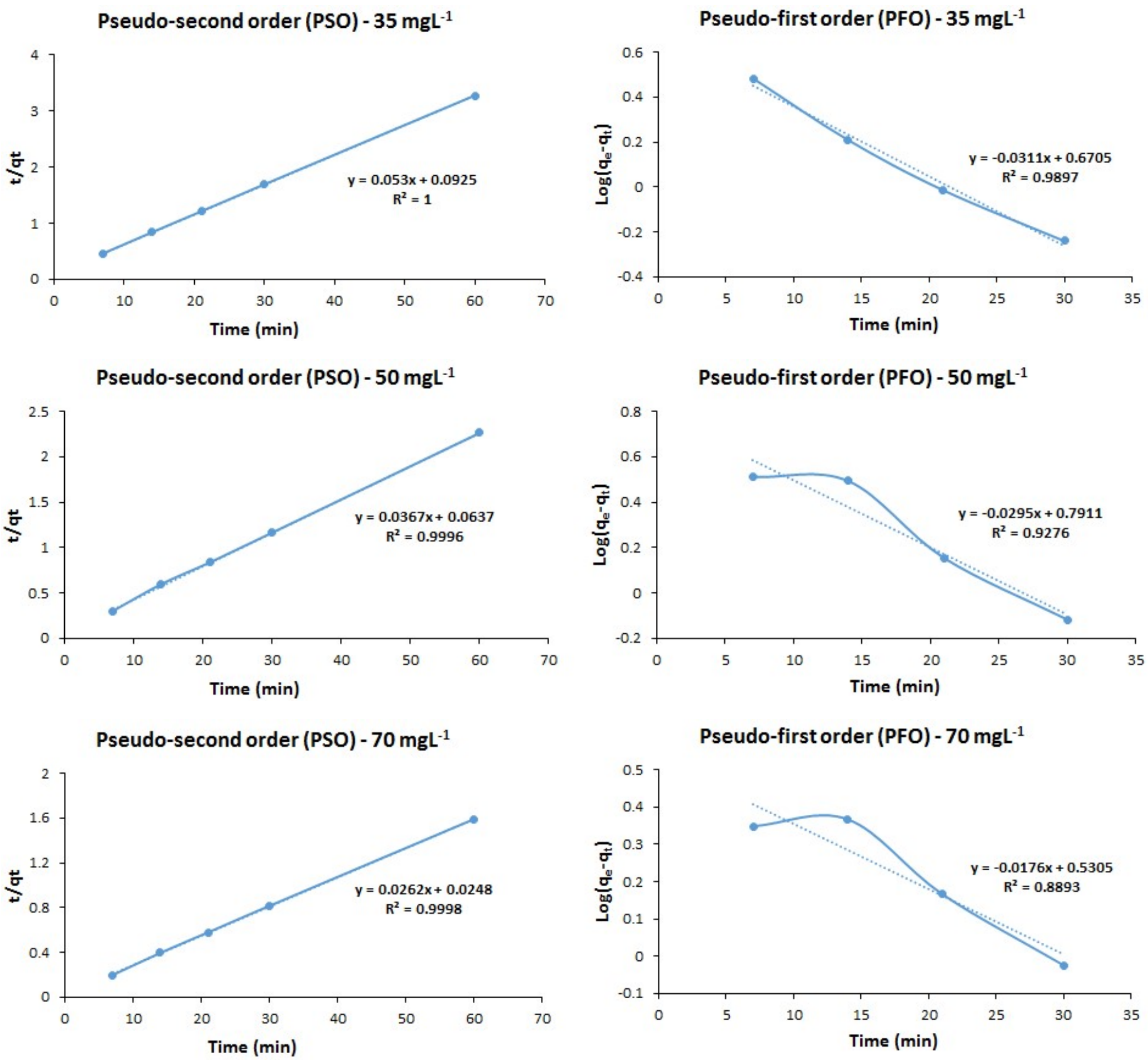

Figure S2. Lineal equation adjusted for the pseudo-first (PFO) and pseudo-second (PSO) kinetics models at different CPX concentrations.

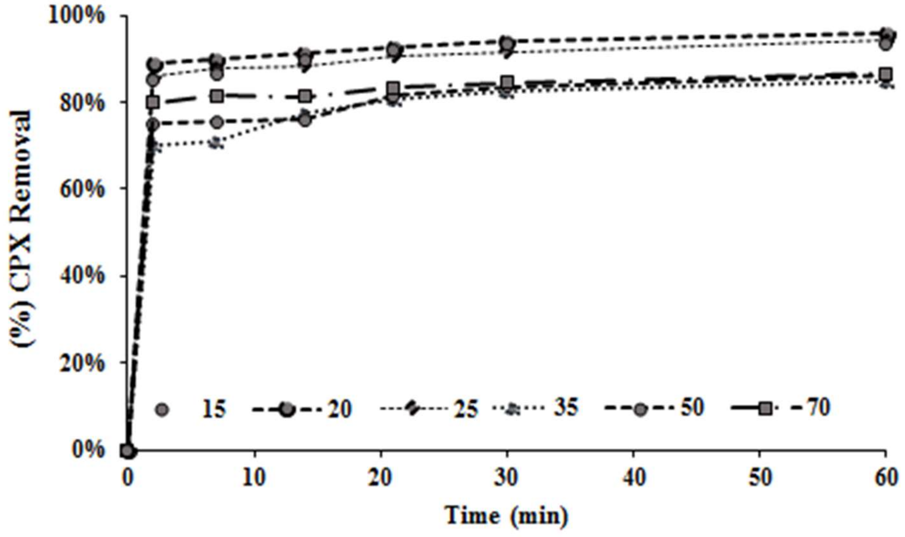

Figure S3. Effect of the initial concentration of CPX on the removal percent removal on F_{Zn}. [Experimental conditions: adsorbent doses: 1.6gL^{-1}; pH: natural]

Figure S3 shows the effect of the initial concentration of CPX on the percentage removal on F_{Zn}. It can be seen that the removal percentage of CPX increased remarkably with an increase in the initial concentration. During the initial step there was a high adsorption rate which was related to the driving force and high concentration Vs the huge amount of active sites. After some time the equilibrium state was reached and there was a low adsorption rate due to the decrease of CPX molecules and binding sites. Similar explanations has been mentioned elsewhere [25][26].

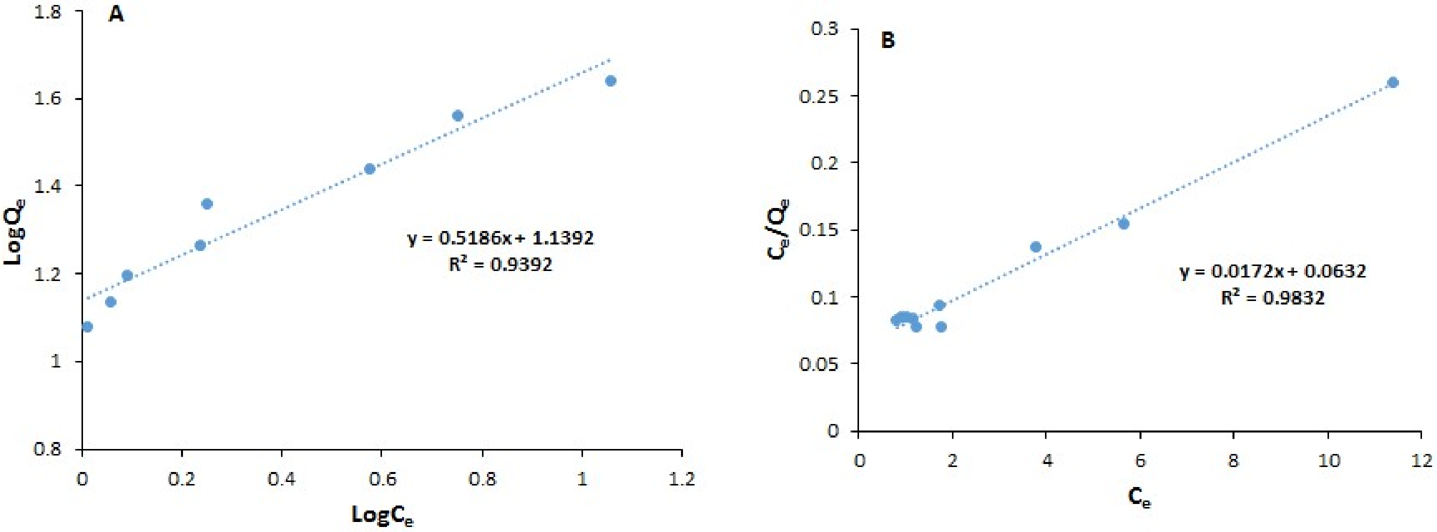

Figure S4. A) Freundlich and B) Langmuir **i**sotherm models for the adsorption of CPX onto F_{Zn} [20 mgL^{-1}, 180 min].

Table S1. Parameters of isotherm models for the adsorption of CPX onto F_{Zn} [20 mgL, 180 min]

**Langmuir**	
Q_m (mgg^{-1})	57.47
k_L (L mg^{-1})	0.2857
R_L	0.1470
Δ Q_e (%)	0.36
R^2	0.984
**Freundlich**	
k_F (mg g^{-1})	13.02
n_F	1.79
Δ Q_e (%)	13.55
R^2	0.940

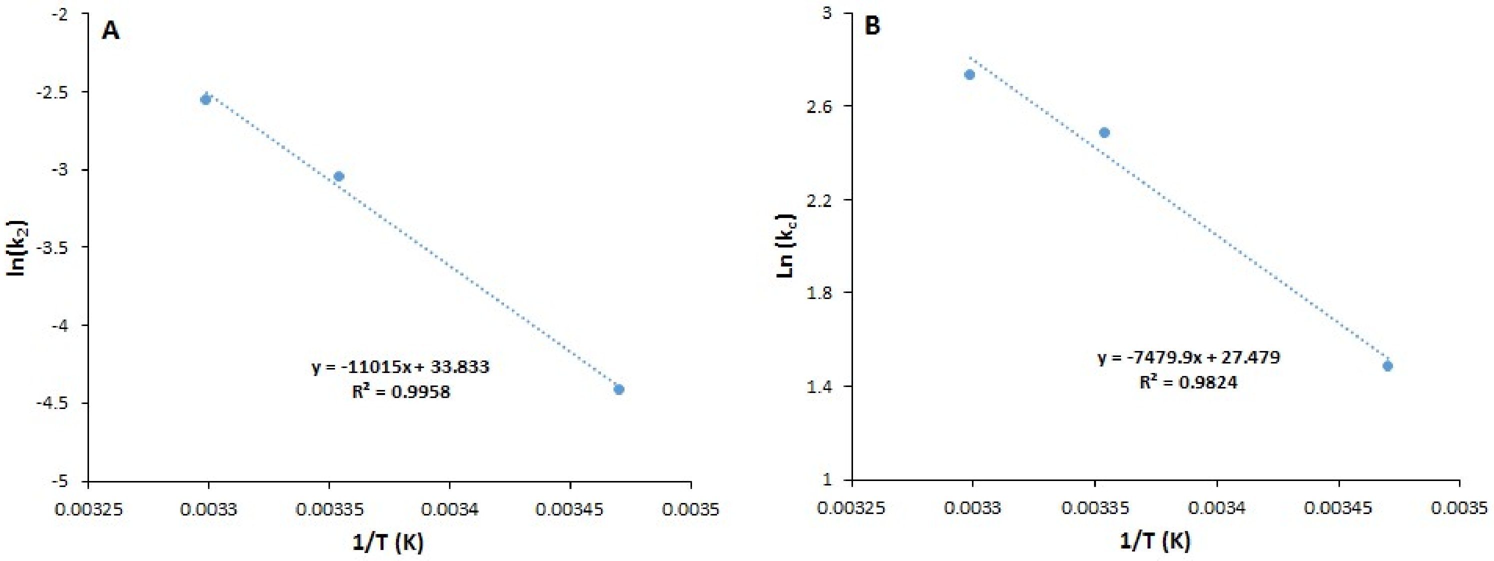

Figure S5. Thermodynamic parameters of CPX adsorption on palm fiber adsorbent **A)** Ea and **B)** Gibbs, Enthalpy and Entropy.

Table S2. Composition of simulated wastewater (pH:6.5) and simulated aged urine (pH:8.5)

Composition WWTP	mg L^{-1}	Composition Aged Urine	g L^{-1}
$NaHCO_3$	96	Na_2SO_4 Anh.	2.3
NaCl	7	NaH_2PO_4 Anh.	2.1
$CaSO_4.2H_2O$	60	NaCl	3.6
Urea	6	KCl	4.2
KCl	4	CH_3COONa	10.21
K_2HPO_4	28	$(NH_4)_2CO_3$	26.59
$CaCl_2.2H_2O$	4		
Peptone	32		
$MgSO_4.7H_2O$	125		
Meat extract	22		
